# Gene Duplication and Phenotypic Changes in the Evolution of Mammalian Metabolic Networks

**DOI:** 10.1371/journal.pone.0087115

**Published:** 2014-01-28

**Authors:** Michaël Bekaert, Gavin C. Conant

**Affiliations:** 1 Institute of Aquaculture, University of Stirling, Stirling, United Kingdom; 2 Division of Animal Sciences, University of Missouri, Columbia, Missouri, United States of America; 3 Informatics Institute, University of Missouri, Columbia, Missouri, United States of America; Georgia Institute of Technology, United States of America

## Abstract

Metabolic networks attempt to describe the complete suite of biochemical reactions available to an organism. One notable feature of these networks in mammals is the large number of distinct proteins that catalyze the same reaction. While the existence of these isoenzymes has long been known, their evolutionary significance is still unclear. Using a phylogenetically-aware comparative genomics approach, we infer enzyme orthology networks for sixteen mammals as well as for their common ancestors. We find that the pattern of isoenzymes copy-number alterations (CNAs) in these networks is suggestive of natural selection acting on the retention of certain gene duplications. When further analyzing these data with a machine-learning approach, we found that that the pattern of CNAs is also predictive of several important phenotypic traits, including milk composition and geographic range. Integrating tools from network analyses, phylogenetics and comparative genomics both allows the prediction of phenotypes from genetic data and represents a means of unifying distinct biological disciplines.

## Introduction

An eventual goal of biology is to integrate our understanding of evolution at the molecular level with that at the phenotypic. One of the most challenging aspects of this problem is what has been referred to as the *genotype-phenotype* map [1], [2]. Even for very simple biological systems such as RNA molecules, genetic distances (*i.e.*, sequence variations) do not map onto phenotypic distances (*i.e.*, secondary structure) in any uniform way [3]. One approach to this question is to statistically associate natural genetic variation to phenotypic variation, through approaches such QTLs [4] or genome-wide association studies [5]. However, these techniques do not define the nature of the association between a trait and its associated genes. Another potential approach is suggested by the new techniques of systems biology, which aims at predicting the behavior of a biological system from the quantitative and dynamic interactions of its components [6], [7]. That there is considerable interest in understanding phenotypes and their evolution is evident from the continued study of the even most fundamental phenotypic traits of organisms (body size, longevity, metabolic rate and body temperature) and of mammalian-specific traits such as milk composition. Among the cellular systems, metabolism is an attractive target for a systems approach because, to a first approximation, the central players are known (at least in model species; [8], [9], [10], [11]). As we will show in this work, metabolism represents one way in which the evolution of phenotypes can be linked to the evolution of their underlying genes.

The study of body size, temperature and metabolism is perhaps best exemplified by the study of the allometric relationships between these variables [12], [13], [14], [15]. In general, researchers have found that metabolic rate scales as a fractional power of the basal metabolic rate, with at least some controversy as to the magnitude of that scaling coefficient [12], [14]. The temperature at which this metabolism occurs (*i.e.*, either body or environmental temperature) also co-varies with body size and metabolic rate, although the direction of causation is not entirely clear [13], [16], [17].

Similarly, aging and longevity are topics of considerable interest to researchers, at least in part for reasons of human self-interest. Experimental work on aging has tended to focus on identifying genes and pathways responsible for, or protective from, aging [18]. One early theory, the *rate of living* hypothesis, proposed an inverse relationship between metabolic rate and lifespan, more or less by analogy to the breakdown of human tools with use. This idea can be made more specific with the proposal that cellular damage may result from side products of metabolism, namely reactive oxygen species, or ROS [18], [19]. Although both of these ideas suggest that lifespan should decrease with increasing metabolic rate, another hypothesis, *uncoupling to survive* makes the opposite prediction [20]. The key difference is the recognition that the strong proton gradients in the mitochondrial intramembrane space that allow high ATP synthesis also increase ROS production. Partly uncoupling proton flow and ATP synthesis increases nutrient use but also reduces ROS production [20]. The relationship of metabolism and aging is also somewhat confused by the observation that caloric restriction tends to increase lifespan [21]. However, there is now evidence that this association is due less to reduction of metabolic rate than to alterations in the patterns of metabolic flux and mitochondrial usage [22].

Naturally, aging and senescence also must be understood as evolutionary phenomena and at least three such theories have been proposed [23]. First, for organisms subject to environment mortality (predation, disease, accident), natural selection cannot act to extend lifespan well beyond the age at which the average individual would be expected to have died due to external causes. Second, there may be evolutionary tradeoffs between early fertility and lifespan, which, coupled with environmental mortality, will also tend to act against selection for long lifespan. Third and related is the *disposable soma* theory, which suggests that metabolic energy devoted to prolonging lifespan comes at the cost of energy not devoted to reproduction and can be selected against [24].

The associations between aging and the allometric traits already discussed suggest the potential for a unification of the evolutionary and genetic theories of aging. The most recent analysis of these variables suggests that lifespan is associated with body size, possibly because of reduced predation on larger individuals, but that there is no independent association of lifespan and metabolic rate once body size is accounted for [25].

No single approach will yield complete understanding of systems as complex as those above, and this work does not attempt to do so. However, new data and approaches that fall under the headings of systems biology and comparative genomics do at least provide a novel perspective on these questions. A particular feature of the *metabolic network* is the large number of isoenzymes (*i.e.*, distinct proteins that catalyze identical reactions). While the existence of isoenzymes has long been known, their evolutionary significance is still unclear. Although not all isoenzymes are products of gene duplication, many are, and possible explanations for their evolutionary persistence include mutational buffering, differential regulation, increased gene dosage, facilitation of evolutionary innovation and functional diversification [26], [27], [28]. Among these possibilities, the importance of *gene dosage* is increasingly appreciated [29], [30], [31], [32]. Thus, in humans, high copy numbers of the starch digesting amylase genes are associated with populations having high-starch diets [33], suggesting a recent increase in the selective benefit of high amylase activity. Such copy number variation contributes significantly to differences in transcript abundance among individuals [34], and some copy number variations are driven to high frequency by positive selection for increased expression of the corresponding gene [33], [35], [36].

We have previously shown that there are considerable numbers of gene duplications and losses in mammalian metabolic networks [37]. Moreover, these copy number alterations (CNAs) are non-randomly distributed in the network and show associations with phenotypic traits of interest such as milk production. In this study, we extend on this work, employing a greatly expanded set of mammalian genomes, a new, phylogenetically-aware mapping procedure and a machine-learning approach to associating phenotypic traits with CNAs. In addition to the traits discussed above (body weight, longevity, metabolic rate and body temperature), we analyze milk characteristics (given our previous results), as well as genomic characteristics (chromosome number and C-value) and habitat.

## Results

### Enzyme orthology network construction

**Figure 1:**
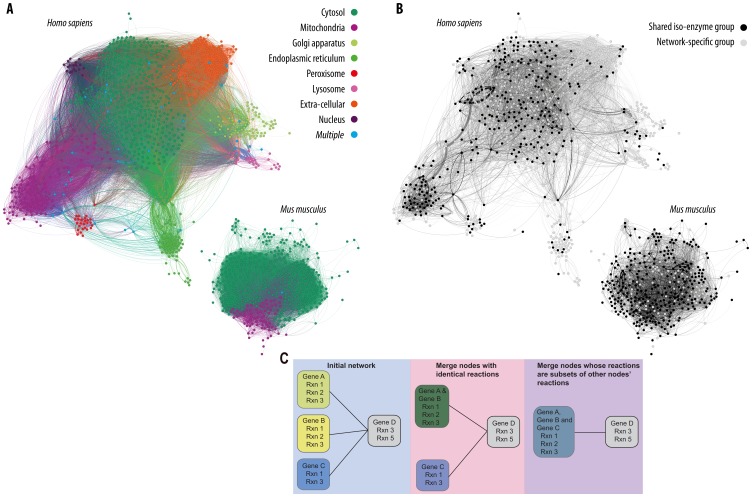
*H. sapiens* and *M. musculus* metabolic networks. (A) Complete metabolic networks with cellular compartments (node and edge colors) are shown. (B) The locations of the shared isoenzyme groups between the networks are illustrated with darkly shaded nodes; pale nodes are those nodes that are not identified in the other network. For both panels currency metabolites have been removed. (C) A cartoon of our approach for creating isoenzyme groups. Genes with identical reaction lists are first merged, followed by a step that combines genes that have only a subset of these reactions. Nodes with overlapping but non-identical reaction lists are not merged.

We used two independent reference metabolic networks in order to provide some level of validation, those of *Homo sapiens* (human) and *Mus musculus* (mouse). The human compartmentalized metabolic network of Duarte *et al*. [8] includes 3,188 metabolites, 3,742 reactions and 1,496 genes. Of the reactions, 2,307 are associated with at least one gene. We previously described a reduction of the metabolic network into classes of enzymes that we refer to as *iso-enzyme groups* [37]. These groups represent sets of enzyme-coding genes involved in either the same reactions or subsets of the same reactions. To create them, we combine network reactions in three steps. We first group genes coding for enzymes that catalyze identical reactions. We then sequentially merge any groups where the reactions of one group are a subset of reactions of a second group. The resulting iso-enzyme groups contain genes that participate in a subset (possibly complete) of the reactions associated with that group. Finally, we define a new type of metabolic network where the nodes are these iso-enyzme groups (Figure 1C). Two such nodes are connected if any of the compounds involved in one node's reactions are shared with the compounds of the other node. Following this procedure, we established 882 isoenzyme groups (Figure 1A): these are the nodes referred to hereafter. The overall network included 4 isolated isoenzyme groups and a main connected component and 71,216 directed edges (as described in the *Methods* section, currency metabolites were removed from the reference networks). Network statistics: diameter: 6, average shortest path: 2.29, density: 0.092. The mouse metabolic network of Selvarasu *et al*. [9] includes 1,288 metabolites, 1,493 reactions and 777 genes. Of the reactions, 1,092 are associated with at least one gene. We defined 413 isoenzyme groups (Figure 1A). The overall network included 2 isolated isoenzyme groups and a main connected component and 20,337 directed edges. Network statistics: diameter: 7, average shortest path: 2.188, density: 0.119. We compared the two networks by mapping reactions from one network onto the other, using the orthologous genes as links. The smaller mouse network shares 70.2% of its nodes with the human network (Figure 1B), while the human network shares 35.5% of its nodes with the mouse. Reactions without assigned genes account for the majority of these differences (data not shown).

Our goal was to study differences in enzyme copy number among eighteen mammalian genomes. To do so, for each of the other seventeen genomes, we first inferred orthologous genes between these genomes and the reference genomes (*H. sapiens* and *M. musculus*) using both sequence homology (inferred with GenomeHistory [38]) and gene order (synteny) data, as described previously [37]. We then used these orthology inferences to map each metabolic network onto each target genome in three steps [37]. First, orthologous genes from the new genome are assigned to the metabolic network nodes of their counterparts in the reference species. Second, any “orphan” genes that are homologous to members of exactly one iso-enzyme group are assigned to that group. Finally, any remaining large gene families for which all annotated members fall into a single iso-enzyme group are also assigned to that group. The resulting mapping between the reference network and the target genome then allows us to identify gene CNAs between the target and reference metabolic networks (e.g., duplications or gene losses in the enzyme-coding genes of one genome relative to a second; Dataset S1).

The metabolic network of Duarte *et al.* [8] is annotated with NCBI gene identifiers. In order to perform our comparative genomics analyses, we translated these identifiers into Ensembl IDs [39]. However, because both the NCBI and Ensembl databases have been updated since our previous analyses, the set of genes retrieved here differs slightly from those given previously [37]. We spent a great deal of time manually refining this mapping step, allowing us to add a few more genes to the network, which in turn resulted in several previously distinct isoenzyme groups being merged. Despite this slight reduction in the number of nodes (from 944 to 882), our current isoenzyme network is very similar to the previous one in terms of global topology: the network density, diameter and average shortest paths are essentially identical (data not shown).

### Ancestral-states networks

**Figure 2:**
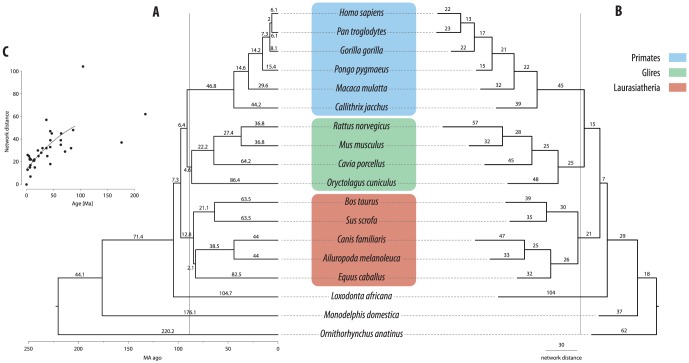
Phylogeny of the species studied. (A) Phylogenetic tree showing the divergence times (in million years) between the species used in this study [40]. The vertical line illustrates a split for these taxa into three large lineages that have similar ages (consistent with the separation observed in Figure 3). (B) Topology of the tree in A, with branch lengths proportional to the network divergence (see *Methods*). Insert (C) Scatter plot of the network divergence vs. divergence times. Regression line is in grey (log-log regression y = 10.86 x^0.3018^, R = 0.72).

The mapping of metabolic networks onto individual genomes (e.g., our enzyme orthology networks) represents only a snapshot of these complex entities. In fact, all eighteen of these networks are related to each other by the phylogeny shown in Figure 2A [40]. To address this fact, we used the established networks to infer the evolutionary history of the CNAs in each isoenzyme group. We used parsimony with the continuous character option to trace all ancestral states on the mammalian phylogenetic tree (Figure 2A). The result is a reconstruction of the ancestral states of each isoenzyme group at all internal nodes in this phylogeny. We define a CNA as any isoenzyme group possessing a different number of included genes in the isoenzyme group as compared to that number in its direct ancestor (Supplementary Data 2). With these reconstructed ancestral networks, we can calculate the degree of network change that has occurred on each branch of the phylogeny, using an averaged distance between the networks at each node in the tree (Figure 2B). This network evolutionary rate (e.g., number of CNAs since a common ancestor) in the enzyme orthology network is related to, but not solely a function of, the divergence times of the species in question (Figure 2C).

### Clustering

We previously found a tendency for CNAs to cluster in the network, so we asked if this result held for these phylogenetically-aware comparisons. For a given pairwise comparison of networks in species A and B (*M_A_* and *M_B_*, respectively), we can, for each node, ask whether the copy-number of the enzymes catalyzing that reaction is the same or different in *M_A_* and *M_B_*. We can then use our previously described tool [37] to detects clusters: this tool works by first removing edges touching nodes that do not show CNAs between *M_A_* and *M_B_*. The tool then calculates connected components among the remaining nodes (which by definition possess CNAs). We assessed the statistical significance of any induced clusters by randomizing the position of these CNAs. When we compared each extant network to its direct ancestor, we did not find, for either network, significantly bigger clusters than would be expected by chance (Table 1). This result is in contrast to our previous analysis, but represents a comparison over a much shorter divergence time (back to the most recent common ancestor with another genome rather than the entire divergence between that species and humans). When we examine the clustering of CNAs between an extant genome and not the most recent ancestor, but two or three ancestors back, there were indeed several genomes showing clustering (Table 1).

**Table 1:** Details of the CNAs clustering analysis.

	Direct ancestor – Species				Ancestor 2 – Species				Ancestor 3 – Species			
	Components		Degrees		Components		Degrees		Components		Degrees	
	Num.	Size	In	Out	Num.	Size	In	Out	Num.	Size	In	Out
*H. sapiens*												
*P. troglodytes*	○											
*G. gorilla*									○	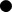	○	
*P. pygmaeus*			○						○	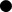	○	○
*M. mulatta*					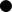	○			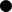	○	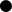	
*C. jacchus*												
*R. norvegicus*	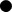	○			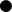	○			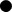	○		
*M. musculus*		○				○			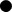	○		
*C. porcellus*												
*O. cuniculus*												
*B. taurus*			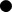	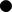			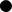				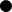	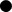
*S. scrofa*					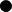						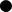	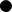
*C. familiaris*												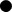
*A. melanoleuca*				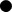							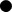	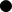
*E. caballus*				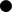			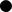	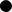			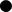	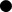
*L. africana*												
*M. domestica*									-	-	-	-
*O. anatinus*	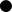	○	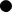		-	-	-	-	-	-	-	-

For each species, an analysis of the CNA was conducted between its current state and that of 1) its direct ancestor, 2) two ancestors and 3) three ancestors back. The results reported include the status of the number and size of the components formed by the CNAs in the enzyme orthology networks and the number (degree) of outgoing (product) and incoming (reactant) metabolites. Bigger/more/higher (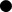); Smaller/fewer/lower (○) at α = 0.05.

### Association of the network structure with diverse traits

**Figure 3:**
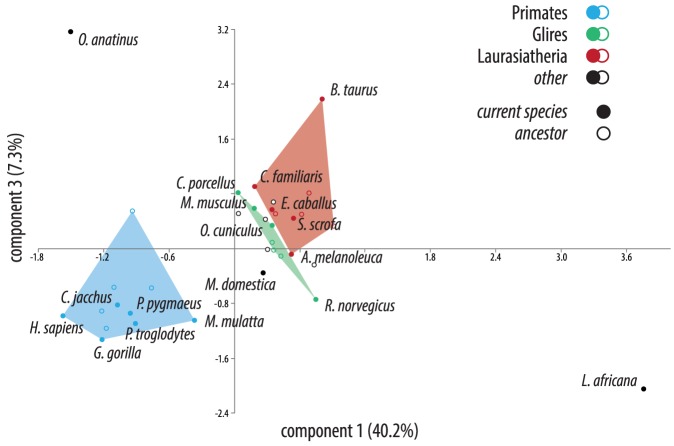
Results of PCA analysis of network CNAs. Components 1 & 3 explain 47.5% of the observed diversity. The three lineages highlighted in Figure 2A/B (including current species and their ancestors) are shaded and show clear separation. Component 1 vs. Component 2 is less interpretable (57.3% of the observed diversity).

Given the inferred networks and the phylogeny, it is possible to explore the role of metabolic changes in the evolution of these diverse mammals. We thus collected a dataset of 17 phenotypic traits (maximum longevity, gestation and weaning times, adult weight, body temperature, metabolic rate, milk composition, C-value, chromosome number, average environmental temperature of the home range, home range precipitation and dispersion from the equator) from these 18 organisms (Table 2 and Table S1). We first used principal component analysis (PCA) to visualize and identify significant differences in these traits and to distinguish which CNAs, if any, were driving those differences (Figure 3). However, based on PCA and subsequent analysis of similarities (ANOSIM), the primary signal appeared to be phylogenetic (separation of primates from other mammals) rather than functional (R = 0.41, *P*≈0.001).

**Table 2:** Trait correlation coefficients and *P*-values.

	*H. sapiens* reference		*M. musculus* reference	
Trait	Pred. correl. coefficient	*P*-value	Pred. Correl. t	*P*-value
Avg. adult weight	0.92	0.03* †	0.70	0.20
C-value	0.93	0.003*	0.78	0.04*
Chrom. number	0.82	0.03*	0.10	0.93
Gestation length	0.41	0.34	0.88	0.004*
Max. dist. equator	0.04	0.95	0.72	0.11
Maximum longevity	0.66	0.07	0.80	0.004*
Metabolic rate/mass	0.98	0.003* †	0.65	0.29
Milk ash	0.68	0.23	0.73	0.12
Milk fat	0.94	0.003*	0.8678	0.004*
Milk lactose	0.64	0.13	0.01	0.99
Milk protein	−0.29	0.61	0.53	0.21
Milk solids	0.88	0.003*	−0.19	0.88
Min. dist. equator	0.90	0.003*	0.86	0.004*
Mean precip.	0.61	0.34	0.89	0.01*
Body temp.	0.49	0.44	−0.02	0.99
Mean env. temp.	0.95	0.003*	0.78	0.10
Weaning	0.49	0.34	0.60	0.20

*Significant *P*-values after controlling for multiple testing (false discovery rate α = 0.05).

† Both weight and metabolic rate/mass vary by more than 10,000-fold over the animals studied, while none of the other traits vary by more than 100-fold. Analyses of the log-transformed weights and metabolic rates suggested that these correlations are not robust to the log –transformation of these data.

The poor performance of PCA suggests that the complex, non-discrete, nature of the traits tested might pose difficulties with standard statistical analyses. We thus adopted a machine learning approach. The data for this approach consists of the number of CNA *events* inferred along each branch of the tree in Figure 2 and the corresponding numerical change in the trait inferred for that branch. The use of changes along each branch ensures phylogenetic independence in our analysis and prevents the pervasive common ancestry in the data from misleading us.

One might think that because both sets of values are drawn based on the same underlying phylogeny that there would be, due to common divergence times, a high correlation in the number of CNAs along a branch and the amount of change in a trait. However, this is not the case: the “branch lengths” taken from CNAs and from traits are rarely highly correlated and occasionally even show significant negative correlations (data not shown). We used least median squares regression (see *Methods*) to compare copy-number changes to the phenotype of interest. This approach allowed us to estimate the correlation coefficient between the trait values and the predictions of those values made using the CNAs (Table 2). We assessed the statistical significance of these correlation coefficients by randomization of the traits among the nodes of the phylogeny and recalculating the associations (see *Methods*). Note that our approach requires that any node selected by the machine learning algorithm must perform well as a predictor when omitting every possible species, meaning that it is predictive regardless of the phylogenetic position of the trait being predicted. In both networks, changes in CNAs in the enzyme orthology network were significantly predictive of the milk fat (*P*≤0.04 after FDR correction). Similarly, there were significant associations between C-value and the minimum distance from the equator (FDR-corrected *P*≤0.004; Table 2). There is also an intriguing correlation between longevity and CNAs in the mouse network, but the association in the human network is non-significant after FDR correction (*P* = 0.07; Table 2).

## Discussion

Using comparative genomics, we have mapped the *H. sapiens* and *M. musculus* metabolic networks onto sixteen other mammalian species, creating enzyme orthology networks for each. In the process, we inferred the set of changes in enzyme copy-number (CNAs) across this phylogeny (Dataset S1). Despite the fact that mammalian genomes vary in both gene content and organization [41], the network topology is relatively conserved across these animals, likely as they are quite closely related relative to other metabolic network comparisons that found greater changes in network structure [42]. Nonetheless, there are reasonably large numbers of CNAs observed (Dataset S2). Having two reference metabolic networks allows us to cross-validate many of our conclusions: even though the reaction coverage in the mouse network is considerably lower, all the analyses provide the same results for both networks (although statistical confidence is sometimes lower for the mouse-derived analyses). Nonetheless, there are caveats to our analyses: many reactions in the two reference metabolic networks either do not require an enzyme to catalyze them or the required enzyme is still unknown. While it is unlikely that these reaction nodes, absent from our enzyme orthology networks, would change our clustering results, it is possible that some of this missing information might have limited the power of our phenotypic comparisons. Likewise, our approach only considers the enzymes common to most or all mammals: if there are species-specific enzymes that adapt a population to a certain environment, our approach would not detect them.

Using this comparative data and the phylogenetic relationships in Figure 2, we also reconstructed the ancestral enzyme orthology networks and the most parsimonious gene copy number for each isoenzyme group at all internals nodes of this tree. These metabolic CNAs are not selectively neutral: they cluster in the network, creating a large interconnected sub-network within the core metabolic network. As we previously found with a subset of these data [37], random distributions of CNAs do not mimic this pattern, indicating that some form of natural selection has acted to preserve duplications (or to favor gene losses) in the network. However, deeper phylogenetic comparisons do not provide significantly stronger associations once one has descended a few nodes in the tree. The fact that CNAs are only loosely associated with the phylogeny suggests that other forces might be driving their evolution.

One such force is selection on phenotypic traits that are products of the metabolic network. We selected a number of potentially associated traits, based on the availability of data in a broad range of organisms and on the potential for a metabolic association. Using a machine-learning approach, we sought to associate each trait with the patterns of CNAs across the enzyme orthology networks and through the phylogeny. We find that we can use pattern of network CNAs in the phylogeny to predict range (minimum distance from the equator), milk chemistry and the genomic C-value. There are also hints that traits like longevity and environmental temperature may show associations, although that association in one network was not significant after FDR correction. Surprisingly, no association was identified between CNAs and either the metabolic rate or the adult body weight. However, we do note that the measurements of these two phenotypic variables were taken by many different researchers over several decades, meaning that all of the values might not be strictly comparable. We were initially surprised at the association between C-value and CNAs, but on reflection recognized that a change in gene duplication rate in a genome would actually, at least at some level, alter both values.

One trait that was not clearly associated with metabolic network structure, longevity, is actually expected to show some relation. Researchers have already sought to move beyond single gene approaches to look at network-based [43], [44] predictors of longevity. The central role of metabolic processes in aging is suggested by a number of lines of evidence, including a positive association between mitochondrial activity and lifespan [22], the potential for reactive oxygen species leaking from the respiratory chain to damage cells and cause aging [18], the observation that caloric restriction induces a number of changes in metabolic regulation and a concomitant tendency toward increased lifespan [45] and the influence of changes in the insulin-signaling pathways on lifespan [46], [47], [48], [49]. More refined metabolic models may show an association between network changes and lifespan that were not evident in our work.

Understanding the nature of the genotype-phenotype map is still one of the most difficult problems in biology. The fact that metabolic network structure is predictive of at least some aspects of phenotype gives insight into the nature of this problem. In particular, the fact that it is the patterns in the network as a whole that are predictive argues again for the importance of genome-scale approaches to understanding biology and suggests the challenges inherent in trying to understand organismal complexity from a strictly “bottom-up” approach.

## Methods

### Data collection and pre-processing

The complete genome annotations for 18 mammals, *Ailuropoda melanoleuca* (giant panda), *Bos taurus* (cow), *Callithrix jacchus* (marmoset), *Canis familiaris* (dog), *Cavia porcellus* (Guinea pig), *Equus caballus* (horse), *Gorilla gorilla* (gorilla), *Homo sapiens* (human), *Loxodonta africana* (elephant), *Macaca mulatta* (macaque), *Monodelphis domestica* (opossum), *Mus musculus* (mouse), *Ornithorhynchus anatinus* (platypus), *Oryctolagus cuniculus* (rabbit), *Pan troglodytes* (chimpanzee), *Pongo pygmaeus* (orangutan), *Rattus norvegicus* (rat) and *Sus scrofa* (pig) were acquired from Ensembl release 60 [39]. For the purposes of homology/orthology assignment, we used the longest transcript for each protein-coding gene, along with its genomic location. We downloaded the *H. sapiens* metabolic network, MODEL6399676120 [8] from the BioModels database [50]. The *M. musculus* metabolic network was obtained from Selvarasu *et al.* [9]. The association between the NCBI gene identifiers in these models and Ensembl gene IDs used for orthology analysis was made with the NCBI gene2ensembl library. We used our previously described orthology inference method [37] to map genes from other genomes onto the human or mouse metabolic network (see Figure 1C). To do so, we have introduced the concept of an *isoenzyme group*. These groups are defined on the basis of sequence similarity and nested metabolic functions [37] and represent the nodes of the enzyme orthology networks employed here. Edges between these nodes are defined by shared metabolites (taken from the reference network) between the included reactions of the pairs of isoenzyme group nodes. The network is directed: for irreversible reactions if the product of one reaction is a reactant in the second, we define a directed edge. Reversible reactions are treated similarly, except that both directions of the reaction are allowed and handled independently (Dataset S1). Thirteen currency metabolites (H^+^, H_2_O, ATP, ADP, Pi, PPi, Na^+^, Co-enzyme A, O_2_, NAD^+^, NADH, NADP^+^, NADPH) were removed from all analyses in every compartment they occurred [51]. We then used the reference networks to locate each metabolite in a cellular compartment.

### Comparing the networks

The gene orthology data from mouse and humans allowed us to map nodes from each network onto the other, allowing us to infer the overlap between the two networks.

### Physical traits

We collected seventeen physical traits for each species (where available): maximum longevity, metabolic rate [52], body temperature [52], [53], milk composition [54], [55], maximal species range (latitude), gestation and weaning times, adult average weight, average environmental precipitation and temperature [52], [56], C-value and chromosome number [57]. *A. melanoleuca* milk composition was taken from Nakamura *et al.* [58] and that of *O. anatinus* from Oftedal and Iverson [59]. When multiple values were available the median was used (Table S1). Free lactose levels were used rather than lactose or sugar composition because the presence of free lactose in the milk is a Eutherian novelty: neither *O. anatinus* nor *M. domestica* produce it [60]. Species with no data for a particular trait had it treated as missing data in that trait's parsimony reconstructions of ancestral nodes.

### Ancestral-states

We used Mesquite v2.73 [http://mesquiteproject.org] to reconstruct the ancestral-state for each isoenzyme group and for the physical markers using the consensus mammalian phylogenetic tree in Figure 2A [40] using continuous-state parsimony.

### Network distance index

We can represent the state of the enzyme orthology network at every node of the phylogeny as a vector *v* with 882 elements (413 for the mouse network, corresponding to the number of nodes or reactions in the network). Each vector element *v*_i_ gives the number of genes associated with that isoenzyme group for that node in the tree. Using these *v*'s, we calculated the Euclidean distance between every pair of nodes *A* and *B* (*e.g.*, end points of all branches) in the phylogeny.

### Principal component analysis

PCA was performed on the covariance matrix of all of the inferred tip CNAs and of the physical traits with the vegan v1.17-8 [61] package for R v2.12.2 [62]. An analysis of similarities [63] was then performed to test the plausibility of the groupings inferred with PCA.

### Machine-learning algorithm

The association between CNAs and absolute gene copy numbers in the enzyme orthology network for each mammal on the one hand and the measured traits for that mammal on the other hand were modeled using the WEKA package [64]. The Least Median Squares algorithm was used; it is a robust linear regression method that minimizes the median (rather than the mean, which might be biased by the non-normal nature of these data) of the squared divergences from the regression line [65]. It repeatedly applies standard linear regression to subsamples of the data and outputs the solution that has the smallest median-squared error [66]. It also replaces missing values (with median values) and re-centers the data. Because irrelevant predictors (here CNAs) have a negative impact on most machine learning schemes, prior to learning, we applied an attribute selection stage that strives to eliminate all but the most relevant CNAs [67]. Thus, the predictive ability of each CNAs individually and the degree of redundancy among them was assessed using the *CfsSubsetEval* algorithm [68] that prefers sets of CNAs that are highly correlated with the variable of interest but have low correlations amongst themselves. Addition of new predictors continues until the prediction quality is no longer improved with the addition of two consecutive predictors, i.e., the BestFirst algorithm [64]. The predictors used considered in the per-node changes in copy number along the branches of the tree in Figure 1 and the target predictions were the corresponding branch-wise trait changes.

Given this set of predictor nodes, we estimate the significance of the association between network structure and traits by sequentially remove each species from the training set and assigning its trait value using the machine-learning algorithm. We then compute the correlation between these assignments and the true values of each trait. The significance of these correlation coefficients was evaluated by comparing them to the distributions of 1,000-reshuffled datasets where the values for the physical marker were randomly reassigned among taxa and the attribute-selection and machine-learning algorithm steps were repeated. As shown in Table 2, the attributes we describe as having significant associations with the enzyme orthology networks have correlations that are significantly higher than that seen among randomized datasets.

### Clustering tests

We used our previously described cluster-detection tool [37]. This approach first removes from the network all nodes without CNAs and then calculates the number of connected components among the remaining nodes. To assess whether these components are bigger than expected, we randomize the position of CNAs in the network and repeat the component calculation. (As an aside, we note that randomization of the *topology* of a metabolic network is a difficult problem [69]: fortunately we need only consider the randomization of CNAs and not of the topology). We can use the distribution of component sizes from 1,000 of these permutations to determine whether the clusters in the real network are larger than expected. The procedure was implemented in C++ using the Boost Libraries [http://www.boost.org/]. The code is available upon request.

## Supporting Information

Table S1 List of reference of the traits. For each trait, the references are provided. Missing data are marked by ‘?’.
(CSV)

Dataset S1 **Inferred enzyme orthology networks (SBML files).**
(GZ) Click here for additional data file.

Dataset S2 **Interactive map of the CNAs for each species (HTML files).** For every node of the mammalian phylogeny, an enzyme orthology network in provided with isoenzyme groups, CNAs and reaction lists.
(GZ) Click here for additional data file.
