# Supplementary material for: Gene Duplication and Phenotypic Changes in the Evolution of Mammalian Metabolic Networks
Source: PLoS One. 2014 Jan 28;9(1):e87115. doi: 10.1371/journal.pone.0087115 (PMC3904969; doi:10.1371/journal.pone.0087115)
Supplement: Dataset S2 — Interactive map of the CNAs for each species (HTML files). For every node of the mammalian phylogeny, an enzyme orthology network in provided with isoenzyme groups, CNAs and reaction lists. (GZ) [file pone.0087115.s003.gz › network/human5.html]

Mammalian networks :: Node 5
